# Development and Validation of a 5K Liquid Chip for Identifying Cashmere Goat Populations in Inner Mongolia Autonomous Region

**DOI:** 10.3390/ani14243589

**Published:** 2024-12-12

**Authors:** Tao Zhang, Qi Xu, Bohan Zhou, Junjie Xiao, Shudan Zheng, Jinquan Li, Qi Lv, Yanjun Zhang, Ruijun Wang, Rui Su, Zhiying Wang

**Affiliations:** 1 College of Animal Science, Inner Mongolia Agricultural University, Hohhot 010018, China; 2 Grassland Research Institute, Chinese Academy of Agricultural Sciences, Hohhot 010018, China; 3 Inner Mongolia Key Laboratory of Sheep & Goat Genetics Breeding and Reproduction, Hohhot 010018, China; 4 Key Laboratory of Mutton Sheep & Goat Genetics and Breeding, Ministry of Agriculture And Rural Affairs, Hohhot 010018, China

**Keywords:** cashmere goats, genetic resource identification, liquid chip, conservation of breeds

## Abstract

(1) Background: Cashmere goats, as one of the characteristic species, are rich in genetic resources. Protecting and rationally utilizing these genetic resources is of great significance for the genetic improvement of cashmere goats. (2) Methods: In this study, tissue samples were collected from *Inner Mongolia white cashmere goats*, which included the Arbas type (*ARBS*); Erlangshan type (*ELS*); Alashan type (*ALS*), *Hanshan white cashmere goats* (*HS*), and *Ujimqin white cashmere goats* (*WZMQ*). Genomic DNA was extracted and subjected to high-depth genome resequencing. GenoBait technology was used for probe design and site optimization, followed by the synthesis of a low-density liquid phase chip. Finally, a total of 281 individuals from five cashmere goat populations in the Inner Mongolia Autonomous Region were randomly selected to verify the chip. (3) Results: The results showed that a total of 5002 SNP sites were finally screened and retained for the synthesis of the low-density liquid chip for identifying cashmere goats in Inner Mongolia Autonomous Region. Principal component analysis and phylogenetic tree construction indicated that the *ARBS*, *ELS*, and *ALS* populations were clustered into one category. (4) Conclusions: This chip can accurately identify the three breeds: *Inner Mongolia white cashmere goats*, *Hanshan white cashmere goats*, and *Ujimqin white cashmere goats*.

## Simple Summary

Cashmere goats, a characteristic species, are rich in genetic resources. Protecting and rationally utilizing these genetic resources is of great significance for the genetic improvement of cashmere goats. In the Inner Mongolia Autonomous Region, there are five nationally recognized populations of cashmere goats: the Arbas type (*ARBS*), Erlangshan type (*ELS*), Alashan type (*ALS*), *Hanshan white cashmere goats* (*HS*), and *Ujimqin white cashmere goats* (*WZMQ*), as well as three breeds: *Inner Mongolia white cashmere goats*, *Hanshan white cashmere goats*, and *Ujimqin white cashmere goats*. This study aims to protect and utilize the genetic resources of *Inner Mongolia‘s cashmere goats* by developing low-density liquid chips. The low-density liquid chip used for identifying cashmere goats in the Inner Mongolia Autonomous Region contains 5002 SNP sites. The sites of this chip are distributed across all chromosomes except the Y chromosome. Most of these SNP sites are located in intergenic and intronic regions. Principal component analysis and phylogenetic tree construction indicated that the *ARBS*, *ELS*, and *ALS* populations cluster into one category, suggesting that the chip cannot accurately distinguish among these three populations. However, this chip can accurately identify the three breeds: *Inner Mongolia white cashmere goats*, *Hanshan white cashmere goats*, and *Ujimqin white cashmere goats*.

## 1. Introduction

As one of the oldest domesticated animals, goats trace their origins back to the region near the Zagros Mountains, with a remarkable breeding history spanning approximately 10,000 years [1]. Integral to the development of human civilization, goats are found worldwide and serve as a crucial resource. They provide not only valued meat and nutritious milk, but also precious fiber, significantly contributing to human sustenance and well-being [2]. In China, there are a total of 78 goat breeds, including 60 local breeds and 18 improved breeds. China has the richest resources of cashmere goats, leading the world in both population and cashmere production. Among these, *Inner Mongolia cashmere goats* and *Liaoning cashmere goats* are important genetic resources in China, renowned for their cashmere quality and yield, respectively. Both breeds serve as paternal sources for the development of cashmere goat breeds in China. The *Inner Mongolia cashmere goats* have been included in the first batch of breeds for genetic resources protection of livestock and poultry in China. Within Inner Mongolia Autonomous Region, there are three cashmere goat breeds, namely *Inner Mongolia white cashmere goat*, *Hanshan white cashmere goat*, and *Ujimqin white cashmere goat*. The *Inner Mongolia white cashmere goats* include three types based on geographical location, the *Inner Mongolia Arbas white cashmere goat*, *Inner Mongolia Erlangshan white cashmere goat*, and *Inner Mongolia Alashan white cashmere goat*. There are notable differences among the cashmere goat breeds, primarily in terms of external characteristics, environmental adaptability, and production performance. The cashmere goats in the Inner Mongolia Autonomous Region are favored by breeders and consumers for their high cashmere production, superior cashmere quality, and delicious meat.

Breed identification in animals involves the application of suitable methods to distinguish individuals of unknown breeds. This process is crucial for the conservation of genetic resources and the enhancement of animal production and reproduction [3]. In the past, due to the limitations of knowledge and technology, breed identification relied primarily on morphological characteristics, such as coat color, body type, ear shape, and other features. For cashmere goats, horn shape was a key criterion. Identifying breeds based on external morphology required an in-depth understanding of the distinctive features of various breeds and extensive experience in classification and breeding, which made this method somewhat restrictive. Moreover, due to the uniformity among animals and the variability in their appearance, this method was prone to significant errors [4,5]. Given the low accuracy of breed identification through external morphology, researchers have turned to physical and chemical indicators, including protein analysis, iris recognition, and infrared spectroscopy technology. Ashoor et al. (1988) successfully identified breeds by analyzing quantitative chromatographic differences in meat slices, achieving promising results. While the accuracy of these technologies has been greatly improved, they remain susceptible to environmental interference and sample quality issues, and the detection process can be cumbersome [6].

Following the 20th century, the rapid development of molecular biology technology has unveiled the potential of DNA molecules in significantly enhancing the precision of animal breed identification. This field encompasses a range of DNA molecular markers, including RFLP, AFLP, SSR, and SNP. In previous studies, microsatellite and ALFP markers were used for such identification [7,8]. However, with the evolution of gene chip technology, SNP loci have become increasingly utilized in breed identification. SNPs are favored for their minimal genotyping error rate, extensive genomic distribution, rapid detection capabilities, and standardization ease. Negrini et al. employed the δ method in breed classification based on the absolute difference in minor allele frequency between breeds [9]. Weir et al. leveraged Wright’s F_ST_ method to discriminate breeds by maximizing allele frequency variances between predefined breed groups [10]. However, these methods are limited to distinguishing between pairs of breeds. Paschou et al. introduced a PCA-based approach to extract informative SNP loci, contributing to the elucidation of population structures [11]. Despite the promotion and application of molecular breeding techniques, the majority of research on *Inner Mongolia cashmere goats* has concentrated on screening candidate genes and analyzing genes expression levels [12,13,14]. The purpose of this study was to develop a low density liquid chip for identification of cashmere goat populations in Inner Mongolia Autonomous Region. This is of great significance for the conservation of cashmere goat breed resources within the region.

## 2. Materials and Methods

### 2.1. Data Collection and SNP Calling

Whole-genome resequencing data were collected from 45 individuals in five cashmere goat populations (*Inner Mongolia Arbas white cashmere goats*, *ARBAS*; *Inner Mongolia Erlangshan white cashmere goats*, *ELS*; *Inner Mongolia Alashan white cashmere goats*, *ALS*; *Hanshan white cashmere goats*, *HS*; and *Ujimqin white cashmere goats*, *WZMQ*) in Inner Mongolia Autonomous Region. Detailed sequencing information can be found in our previous study [15]. According to the outcomes of principal component analysis (PCA) and evolutionary tree analysis, the resequencing data from three individuals (*ELS-5*, *ALS-2*, and *ALS-9*) were removed, leaving data from 42 individuals for site screening. To ensure the quality of the SNPs, vcftools (v0.1.16) was used to filter all SNPs [16]. The filtering criteria were as follows: sequencing depth ≥ 5X, missing rate < 10%,heterozygosity rate < 30%.

### 2.2. Probe Design and Selection of Specific SNP Sites 

After quality control, high-quality SNPs were selected based on the following criteria: a length of 110 base pairs, a GC content ranging from 30% to 70%, and no more than five homologous regions. For SNPs that met these conditions and complied with probe design principles, minor allele frequency (MAF) values were calculated for each site across the five cashmere goat populations.

Additionally, the ΔMAF value, representing the difference in MAF between two populations, was determined for each site. The 1500 SNP sites with the highest △MAF value were selected from each population, with duplicate loci removed. This process resulted in a total of 7298 SNP sites being retained. Considering the probe panel capacity and the excess of sex chromosome sites, all autosomal sites and the top sites with the highest mean ΔMAF for sex chromosomes across the five cashmere goat populations were selected. Initially, 6000 sites were retained for testing and analysis. According to the detection results, some sites with low detection rates were deleted, and ultimately, 5002 SNP sites were kept. The probe and chip synthesis tasks were completed by MOLBREEDING Biotechnology Co., Ltd. (Shijiazhuang, China). The ANNOVAR software (version 3) was utilized to perform gene functional annotation for the sites in this chip [17].

## 3. Validation of 5K Liquid Chip

### 3.1. DNA Collection and Library Construction

In order to further verify the reliability of the genetic resource identification chip for five cashmere goat breeds in Inner Mongolia Autonomous Region, we tested 300 qualified cashmere goat DNA samples. The samples were from 60 individuals in each population. The genomic DNA was evaluated using 1% (*w*/*v*) agarose gel electrophoresis, and its quantity was determined with the Qubit 2.0 Fluorometer (Invitrogen, Shanghai, China). Genomic DNA libraries were prepared utilizing the GenoBauts DNA-seq Library Prep Kit (MolBreeding Biotechnology Co., Shijiazhuang, Hebei, China), adhering to the manufacturer’s guidelines. These libraries were then hybridized with biotin-labeled target probes at 65 °C for 16 h. To capture the hybridized targets and eliminate non-target fragments, Dynabeads MyOne Streptavidin C1 and binding buffer were employed. The captured fragments were subsequently amplified using library amplification primers and DNA polymerase. After two rounds of purification with Beckman AMPure Beads, the libraries were re-quantified using the Qubit 2.0 Fluorometer (Invitrogen, Shanghai, China).

### 3.2. Sequencing, Quality Control, and Variant Detection

DNA samples were sequenced using the 5K liquid chip. The raw reads obtained by sequencing were for quality control. Reads containing adaptor sequences and those with more than 10% N base content were removed. Additionally, if the proportion of bases with a quality score below 5 in any read exceeded 50% of the total read length, the corresponding paired reads were removed. The Burrows–Wheeler Alignment software (BWA v0.7.17), with parameters MEM-T4-K32-m, was used to align the cleaned reads to the assembled high-quality reference genome utilized by our research team [18]. According to the alignment of clean reads to the reference genome, the Unified Genotyper module in GATK (4.2.6.1) toolkit was applied to detect genetic variants [19]. Then, the Variant Filtration module was used to refine the detected variants.

### 3.3. Principal Component Analysis and Phylogenetic Tree Construction

The GCTA software (1.94.0beta) was employed to conduct principal component analysis (PCA) on the SNPs of all samples across the five cashmere goat populations using the command gcta64 --grm cashmeregoats_grm --pca 3 --out cashmere goats_pca [20].The three largest eigenvectors generated by PCA analysis were taken as the principal axes, and the plot function in R 4.3.3 was used to draw PCA plots [21]. The genetic distances among populations, based on the filtered high-quality SNPs, were calculated, and then, the Neighbor-Joining (NJ) method was applied with TreeBest software (version 7)to construct a phylogenetic tree of the SNPs. Finally, the population phylogenetic tree was visualized on the itol website (https://itol.embl.de/).

## 4. Results

### 4.1. Determination of Specific SNP Sites in the Five Cashmere Goat Breeds 

After filtering the resequencing data from 42 individuals, a total of 110,713 high-quality SNPs were obtained. Further probe design was carried out, and the probes of 72,319 SNPs were successfully designed. Taking into account factors such as the maximum ΔMAF value, panel probe capacity, the prevalence of sex chromosome sites, and poor detection rates for sites that complied with probe design principles, 5002 SNP sites were ultimately reserved for the chip synthesis.

### 4.2. The Distribution of SNP Sites on Chromosomes for the 5K Liquid Chip

**Figure 1:**
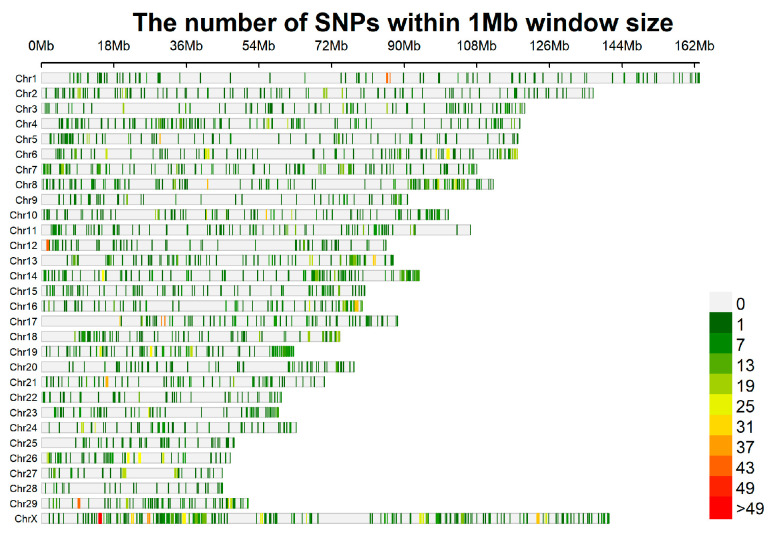
The distribution of SNP sites in chromosomes.

**Figure 2:**
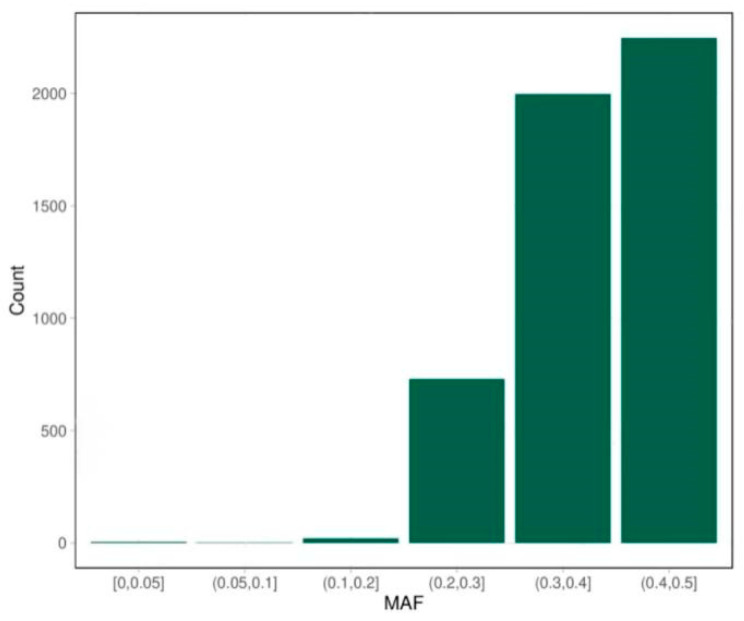
Frequency distribution of MAF of sites in the 5K chip.

For the 5K chip, a 1 Mb window was used to slide along chromosomes, tallying the SNPs within each segment. The distribution and number of SNP sites on each chromosome are shown in Figure 1. Excluding the sex chromosomes, chromosome 8 exhibited the highest density of SNP sites, while chromosome 28 had the sparsest distribution. It was also observed that the number of core regions on each chromosome aligned with the SNP count, suggesting that the final customized chip captured precisely one SNP site per probe. The minimum allele frequency (MAF) distribution of the SNP sites on the 5K chip is presented in Figure 2. A majority of the MAF values are concentrated between 0.2 and 0.5, with 99.4% of the sites falling within this range. Notably, only 28 SNP sites have MAF values below 0.2.

### 4.3. Annotation of SNPs in the 5K Liquid Chip

**Figure 3:**
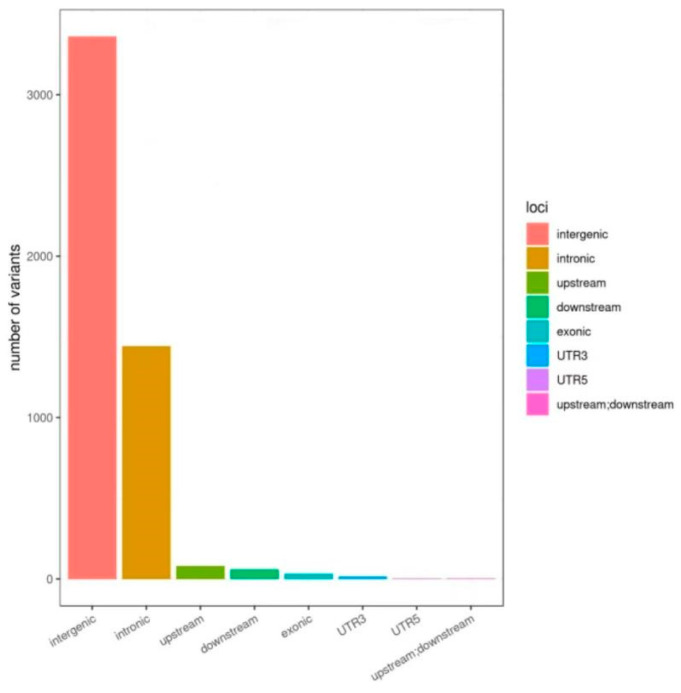
Annotation of all the sites in the 5K chip. Note: (1) Intergenic: The variation is annotated in an intergenic region. (2) Intronic: The variation is annotated in the intron region. (3) Exonic: The variation is annotated in the exon region. (4) Downstream: The 1 Kb downstream region of the gene. (5) Upstream: The 1 Kb upstream region of the gene. (6) UTR3: untranslated area at the 3′ end. (7) UTR5: 5′ end untranslated area. (8) Upstream; downstream: both 1 Kb upstream of one gene and 1 Kb downstream of another gene.

The gene structure distribution for all SNP sites included in the 5K chip is shown in Figure 3. The majority of these sites are found in the intergenic and intronic regions, with 3361 SNPs in the intergenic region and 1442 SNPs in the intronic region, collectively accounting for 96.0% of the total SNP sites. Within the exon region, there are 32 SNPs identified. Additionally, 86 SNPs are situated in the upstream 1 Kb region, and 65 SNPs in the downstream 1 Kb region of genes. The numbers of SNPs in the 5′ and 3′ untranslated regions (UTRs) are 4 and 16, respectively. Intriguingly, there are a total of four SNPs in the 5′ UTR of one gene and the 3′ UTR of another gene.

### 4.4. Validation of the 5K Liquid Chip for Identification of Five Cashmere Goat Populations

In this study, a total of 300 DNA samples were used to evaluate the performance of the 5K liquid chip. Upon evaluating the DNA samples, seven were deemed unqualified and 12 that had been used for preliminary testing were ultimately excluded. Consequently, 281 individuals were retained for identification and verification across five Inner Mongolia cashmere goat populations. The detailed sample information for validation is shown in Table 1. Among the retained samples, two exhibited a low sequencing detection rate due to library preparation issues. The calling rate for the remaining samples ranged from 99.06% to 99.98%, with an overall average detection rate of 99.77%.

**Figure 4:**
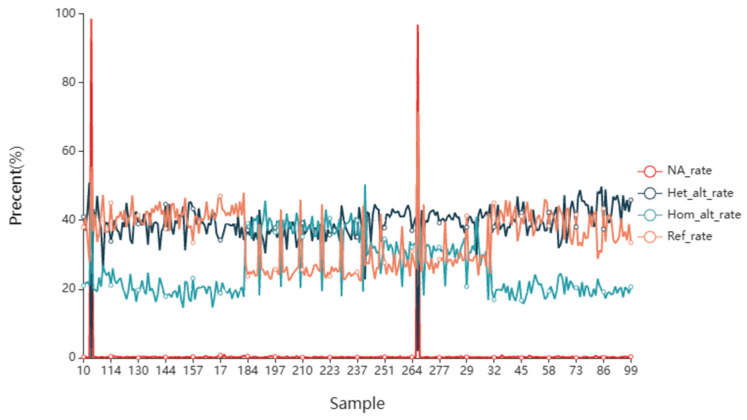
SNP site mutation statistics.

The missing and mutation rates of SNPs in each sample are shown in Figure 4 and detailed in Supplementary Table S1. Notably, two individuals stood out with exceptionally high missing rates, at 96.84% and 98.56%, respectively. For the remaining samples, the missing sites varied from 1 to 47, with an average missing rate of 0.23%. The range of homozygous mutation sites was 728~2516, with an average homozygous mutation rate of 26.20%. The heterozygous mutation sites ranged from 1135 to 2549, with an average heterozygous mutation rate of 39.09%. The range of sites consistent with the reference genome was 1115–2386, with an average consistency rate of 34.47%.

The statistical analysis of missing rates among the five cashmere goat populations is presented in Table 2. It can be found that there are minimal differences in missing rates across these populations. The missing rate of *Ujimqin white cashmere goats* is the lowest, while those of *Inner Mongolia cashmere goats* (Alashan type) and *Inner Mongolia cashmere goats* (Arbas type) are the highest. In terms of heterozygous mutation rates, the Inner Mongolia cashmere goat (Erlangshan type) population shows the highest, contrasting with the *Hanshan white cashmere goat* population, which has the lowest. Conversely, the homozygous mutation rate is highest in the *Hanshan white cashmere goat* population, and lowest in the *Inner Mongolia cashmere goats* (Arbas type). The consistency rate with the reference genome is found to be the lowest in the *Hanshan white cashmere goat* population, and the highest in the *Inner Mongolia cashmere goats* (Arbas type).

### 4.5. Principal Component Analysis and Phylogenetic Tree Construction

**Figure 5:**
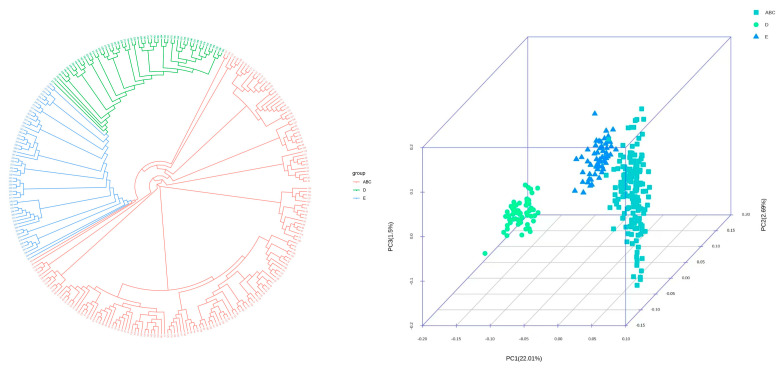
Phylogenetic tree and principal component analysis. Note: ABC: *Inner Mongolia cashmere goats* (including Arbas type, Alashan type, and Erlangshan type); D: *Hanshan white cashmere goats*; E: *Ujimqin white cashmere goats*. Left: Phylogenetic tree for the three cashmere goat breeds; Right: Principal Component Analysis for the three cashmere goat breeds.

This study fully utilized the 5K liquid chip sequencing data from 279 cashmere goats across five populations in Inner Mongolia Autonomous Region to conduct principal component analysis and to construct a phylogenetic tree. The results are shown in Figure 5. From this, it can be seen that the three types of *Inner Mongolia cashmere goat*, including Arbas type, Erlangshan type, and Alashan type, are grouped together (designated as ABC), indicating relatively close genetic distances. At present, it is difficult to distinguish these three populations using the 5K liquid chip alone, and further careful screening of loci information for each population is required to facilitate the identification of the three Inner Mongolia cashmere goats types. However, the 5K liquid chip has proven effective for accurate population identification among the following three breeds: *Inner Mongolia cashmere goats*, *Hanshan white cashmere goats*, and *Ujimqin white cashmere goats*.

## 5. Discussion

The identification of animal breeds is crucial for the protection of genetic resources and the development of new breeds. The main techniques employed for breed identification include microarray chips and targeted capture sequencing, also known as “liquid phase chips” or genotyping by target sequencing (GBTS) [22]. Niu Anran et al. (2022) used SNP chips to distinguish between Duroc, Large White pigs, and other pig breeds. They successfully identified individuals from the core population of these three breeds by using PCA and population genetic structure analysis, offering excellent genetic resources for the future development of specialized lines and composite lines [23]. Min B R et al. (1995) used random amplified polymorphic DNAs (RAPDs) to differentiate among Korean cattle beef, Holstein beef, and imported beef [24]. Min et al. (1995) identified beef breeds using random amplified polymorphic DNAs [24]. Miao et al. (2023) developed a web tool for the global identification of pig breeds [25]. With advancements in sequencing technology, cost-effective and low-density SNP chips have been used as a reliable method for genetic resource identification, aiding in the conservation efforts, and facilitating the tracing of germplasm sources.

At present, several studies have reported on the use of chips for goat breeding purpose. However, no specialized chip exists specifically for the identification of goat breeds. Tosser et al. pioneered the development of a 52K medium-density goat SNP chip utilizing sequencing data from 97 individuals across six goat breeds [26]. But this chip lacks the inclusion of sequencing data from goats in China, leading to potential inaccuracies in genomic selection, genome-wide association analysis of the economic traits, and genetic resource identification for Chinese goat populations. Qiao Xian et al. (2017) expanded on this by developing a goat 66K SNP capture chip, incorporating resequencing data from domestic breeds such as *Inner Mongolia cashmere goats* (*Arbas* type, *Erlangshan* type, *Alashan* type) and *Liaoning cashmere goats* [27]. China has abundant genetic resources in goats and variability among breeds. In order to improve the availability of commercial SNP chips for goats, Wang Fenghong et al. (2021) used resequencing data from 598 individuals representing 85 goat breeds globally and integrated single nucleotide polymorphisms (SNPs) that significantly affect important economic traits, adaptability, and diseases in goats. Consequently, they designed a 70K goat SNP chip [28]. Currently, it has been widely adopted in goat breeding programs.

Thus, it can be seen that multiple commercial goat chips have been released, but there is a notable absence of chips specifically designed for the protection of genetic resources in cashmere goats. This study marks the first instance of screening core SNPs with high inter-population variation frequencies based on resequencing data for population identification, significantly reducing sequencing costs and enhancing the accuracy and convenience of goat breed identification. In line with the design principles of previous chips, the primary criteria for SNP selection are the uniformity of sites and the frequency of SNP variation. Uniformity of SNP sites ensures relatively consistent signal strength across various regions of the chip, thereby minimizing errors in chip manufacturing and data analysis. The mutation frequency of SNPs can impact the detection rate of mutation sites. Therefore, different methods are adopted for SNP site selection depending on the purpose of the chip. Wang Yanyun et al. have identified carp breeds using chip data, and in the screening of SNP sites for this chip, it is crucial to ensure a sufficiently large difference in minor allele frequency (MAF) between breeds [29]. This approach is fundamentally consistent with the chip design concept of this study.

This study strictly selected SNP sites to develop a low-density chip aimed at identifying the genetic resources of cashmere goats in the Inner Mongolia Autonomous Region. The individual DNA samples from the selected cashmere goat populations were obtained from purebred individuals within their respective conservation farms. High-depth resequencing was conducted on 45 individuals from five goat populations in Inner Mongolia Autonomous Region, and the SNP data underwent strict filtering. Individuals with poor clustering performance were excluded based on filtered information and subsequent evolutionary tree analysis, ensuring the uniqueness and accuracy of SNPs for chip design. Firstly, SNP sites with higher minor allele frequency (MAF) were selected to guarantee that variations could be effectively detected in subsequent analyses. On this basis, SNPs with larger inter-breed MAF differences were chosen, and those meeting both criteria were designated as the core sites of the chip. Unrelated background sites were removed, and the total number of sites was reduced to achieve breed identification, thereby reducing the cost associated with using the chip. According to the above method, a total of 5002 core sites were finally retained for inclusion on the chip. Generally, the length of a chromosome is expected to correlate with the number of SNPs on the chromosome, with SNPs being spaced at appropriate intervals. However, in this study, the numbers of SNPs on certain chromosomes (1, 5, 9, 11, 15) were not directly proportional to the length of those chromosomes. Imposing strict uniformity requirements for alignment points might diminish the accuracy of variety identification, so this study did not impose too many restrictions on the uniformity of SNPs in this chip. This is the reason for the uneven ratio of the number of SNPs on each chromosome in the selected SNPs of the chip to the length of the chromosome.

To further evaluate the efficacy of the 5K liquid chip, genotyping was performed on 281 individuals from five cashmere goat populations in Inner Mongolia Autonomous Region, followed by a population difference analysis. The average detection rate for SNPs was found to be 99.77%, suggesting that the chip has good performance, slightly surpassing the detection rate of chips designed for pig breed identification [30]. All SNPs selected for this chip had a coverage of over 5X, with the highest exceeding 100X, making this 5K liquid chip suitable for identifying cashmere goats in Inner Mongolia Autonomous Region. To further verify the effectiveness of the chip, random samples from five cashmere goat populations were genotyped to gather comprehensive core sequencing data, which were then used for principal component analysis and phylogenetic tree construction. The findings indicated that the three Mongolia cashmere goat populations cluster closely together, suggesting that the 5K liquid chip has limitations in distinguishing among these three populations.

A more detailed consideration of the SNP site information for these three populations is necessary to rescreen loci for population identification. However, the chip can be effectively used for precise population identification of three cashmere goat breeds in Inner Mongolia Autonomous Region, including *Inner Mongolia white cashmere goats*, *Hanshan white cashmere goat* and the *Ujimqin white cashmere goats*. It was indicated that the SNP site selection for the 5K liquid chip was stringent and reliable. Compared to the principal components and evolutionary tree results from resequencing, the SNP sites selected for this chip were more specific and representative of the breeds, enabling accurate identification of the three cashmere goat breeds in Inner Mongolia Autonomous Region.

## 6. Conclusions

This study fully utilized high-depth resequencing data from five cashmere goat populations in Inner Mongolia Autonomous Region to identify polymorphic loci among populations. After a rigorous screening process, 5002 SNP sites were ultimately selected to synthesize a liquid chip for the identification of cashmere goat germplasm resources in Inner Mongolia Autonomous Region. The 5K liquid chip is capable of accurately identifying three distinct breeds: *Inner Mongolia cashmere goats*, *Hanshan white cashmere goats*, and *Ujimqin white cashmere goats*. However, it falls short in accurately discriminating between the three populations of *Inner Mongolia cashmere goats*, highlighting the need for further refinement in SNP site selection for more precise population-level identification.

## Figures and Tables

**Table 1:** Sample information for cashmere goats in each population.

Name	Sex	No.	Stock
*ARBS*	female	56	Inner Mongolia Yiwei White Cashmere Goat Co., Ltd. (Ordos, China)
*ELS*	female	57	Inner Mongolia Mizhen International Trade Co., Ltd. (Bayannaoer, China)
*ALS*	female	54	Alxa Left Banner Goat Breeding Farm
*HS*	female	56	Chifeng Bahrain Right Banner Hanshan Goat Breeding Farm
*WZMQ*	female	58	Ujimqin White Goat Original breeding Farm

Note: *ARBAS*: *Inner Mongolia cashmere goats* (Arbas type); *ELS*: *Inner Mongolia cashmere goats* (Erlangshan type); *ALS*: *Inner Mongolia cashmere goats* (Alashan type); *HS*: *Hanshan white cashmere goats*; *WZMQ*: *Ujimqin white cashmere goats*.

**Table 2:** Statistics of SNP site mutation.

Population	Number of Missing Sites	Missing Rate	Number of Heterozygous Sites	Heterozygous Mutation Rate	Number of Homozygous Sites	Homozygous Mutation Rate	Consistent Number of Sites	Site Consistency Rate
*ARBAS*	12	0.25	1954	39.16	968	19.40	2068	41.45
*ELS*	12	0.24	2095	41.97	1044	20.92	1851	37.10
*ALS*	12	0.25	1938	38.85	974	19.52	2077	41.63
*HS*	11	0.23	1815	36.36	1937	38.81	1239	24.83
*WZMQ*	11	0.21	2022	40.51	1592	31.89	1378	27.60
Mean	12	0.23	1965	39.37	1303	26.11	1723	34.52

Note: *ARBAS*: *Inner Mongolia cashmere goats* (Arbas type); *ELS*: *Inner Mongolia cashmere goats* (Erlangshan type); *ALS*: *Inner Mongolia cashmere goats* (Alashan type); *HS*: *Hanshan white cashmere goats*; *WZMQ*: *Ujimqin white cashmere goats*.

## Data Availability

The original contributions presented in the study are included in the article/Supplementary Materials, further inquiries can be directed to the corresponding authors.

## Supplementary Materials

The following supporting information can be downloaded at: https://www.mdpi.com/article/10.3390/ani14243589/s1, Table S1: SNP detection results with the 5K liquid chip.
