# Supplementary material for: Development and Validation of a 5K Liquid Chip for Identifying Cashmere Goat Populations in Inner Mongolia Autonomous Region
Source: Animals (Basel). 2024 Dec 12;14(24):3589. doi: 10.3390/ani14243589 (PMC11672763; doi:10.3390/ani14243589)
Supplement: Supplementary file 1 [file animals-14-03589-s001.zip › animals-3305124-supplementary.pdf]

Supplemental Table S1 SNP detection results with the 5K liquid chip

Sample	NA_number	NA_rate(%)	Het_alt_number	Hom_alt_number	Ref_number
10	15	0.30	2045	1048	1894
100	16	0.32	1844	1082	2060
101	5	0.10	2122	1101	1774
102	6	0.12	2545	1054	1397
103	4930	98.56	0	32	40
105	18	0.36	2154	1000	1830
106	14	0.28	1922	985	2081
108	8	0.16	2352	963	1679
109	5	0.10	2028	1170	1799
11	14	0.28	1875	1029	2084
110	12	0.24	1284	1341	2365
111	4	0.08	1928	1241	1829
112	13	0.26	1971	1193	1825
113	6	0.12	1903	1246	1847
114	21	0.42	1687	1051	2243
115	32	0.64	1832	1294	1844
117	15	0.30	1996	1122	1869
118	20	0.40	1858	1076	2048
119	10	0.20	1827	1129	2036
12	14	0.28	1709	1157	2122
120	6	0.12	1970	1031	1995
124	5	0.10	1899	950	2148
125	18	0.36	2245	848	1891
126	13	0.26	2008	970	2011
127	8	0.16	1711	1218	2065
128	14	0.28	1655	978	2355
129	11	0.22	2074	1000	1917
13	12	0.24	2121	812	2057
130	2	0.04	1945	985	2070
131	18	0.36	2002	1000	1982
133	17	0.34	1918	1123	1944
134	19	0.38	2086	969	1928
135	13	0.26	1948	920	2121
136	15	0.30	1638	1237	2112
137	6	0.12	2104	973	1919
138	19	0.38	1809	1007	2167
139	15	0.30	1951	933	2103
14	19	0.38	1965	807	2211
140	5	0.10	1942	920	2135
141	14	0.28	1558	1162	2268
142	6	0.12	1844	1070	2082

143	18	0.36	1898	945	2141
144	12	0.24	2228	891	1871
145	5	0.10	1843	919	2235
146	7	0.14	2233	922	1840
147	10	0.20	1891	879	2222
148	7	0.14	1791	959	2245
149	13	0.26	1991	976	2022
15	7	0.14	1952	872	2171
150	15	0.30	2069	1058	1860
151	2	0.04	2044	840	2116
152	8	0.16	2198	728	2068
153	11	0.22	1559	1110	2322
154	16	0.32	1951	1087	1948
155	10	0.20	2254	852	1886
156	17	0.34	2255	816	1914
157	16	0.32	2162	1154	1670
158	5	0.10	2109	846	2042
159	5	0.10	1932	1001	2064
16	9	0.18	2171	1020	1802
160	9	0.18	1951	1012	2030
161	5	0.10	1971	1038	1988
162	22	0.44	1915	1025	2040
163	24	0.48	1787	927	2264
164	7	0.14	1954	1036	2005
165	13	0.26	1924	1014	2051
166	7	0.14	2107	730	2158
167	7	0.14	2097	881	2017
168	13	0.26	1808	1115	2066
169	26	0.52	1730	935	2311
17	38	0.76	1698	932	2334
170	3	0.06	1752	1092	2155
171	47	0.94	1840	999	2116
172	4	0.08	1968	966	2064
173	7	0.14	1944	939	2112
174	20	0.40	1920	1008	2054
175	16	0.32	1915	854	2217
176	11	0.22	1951	959	2081
177	9	0.18	1773	981	2239
178	8	0.16	2076	872	2046
179	11	0.22	1739	985	2267
18	17	0.34	1896	1024	2065
180	33	0.66	1702	881	2386
183	23	0.46	1766	1857	1356
184	24	0.48	1849	1948	1181

185	8	0.16	1776	1966	1252
186	5	0.10	2051	1726	1220
187	17	0.34	1696	2022	1267
188	18	0.36	1815	1985	1184
189	7	0.14	1718	2071	1206
19	3	0.06	2153	904	1942
190	6	0.12	1801	1894	1301
191	5	0.10	1711	2014	1272
192	15	0.30	1514	2278	1195
193	9	0.18	1882	1847	1264
194	22	0.44	1787	2025	1168
195	26	0.52	1846	1994	1136
196	18	0.36	1872	1827	1285
197	18	0.36	1885	1819	1280
198	4	0.08	1749	1989	1260
199	22	0.44	1799	1980	1201
20	3	0.06	1818	1030	2151
201	9	0.18	1753	1999	1241
202	3	0.06	1874	1865	1260
203	24	0.48	1567	2090	1321
204	7	0.14	1984	1888	1123
205	7	0.14	1868	1973	1154
206	9	0.18	1982	1797	1214
207	8	0.16	1893	1848	1253
208	24	0.48	1594	2097	1287
209	11	0.22	1727	1948	1316
21	5	0.10	1684	1015	2298
210	7	0.14	1957	1758	1280
211	13	0.26	1791	2007	1191
212	7	0.14	1837	1841	1317
213	8	0.16	1891	1906	1197
214	1	0.02	1941	1877	1183
215	4	0.08	1696	2062	1240
216	19	0.38	1877	1769	1337
217	13	0.26	1876	1760	1353
218	8	0.16	1716	1994	1284
219	14	0.28	1938	1798	1252
22	18	0.36	1794	965	2225
220	5	0.10	1926	1842	1229
221	7	0.14	1686	2153	1156
222	9	0.18	1866	1882	1245
223	8	0.16	1784	2025	1185
224	11	0.22	1822	1844	1325
225	7	0.14	1948	1797	1250

226	11	0.22	1877	1808	1306
228	9	0.18	1856	1891	1246
229	9	0.18	1697	2017	1279
23	17	0.34	2210	890	1885
230	12	0.24	2026	1829	1135
231	4	0.08	1903	1947	1148
232	15	0.30	1826	1951	1210
233	12	0.24	1771	1982	1237
234	9	0.18	1708	2103	1182
235	9	0.18	1995	1781	1217
236	11	0.22	1726	2037	1228
237	11	0.22	1751	1994	1246
238	7	0.14	2190	1668	1137
239	12	0.24	1961	1914	1115
24	10	0.20	1829	962	2201
240	8	0.16	1139	2516	1339
243	9	0.18	2119	1490	1384
244	7	0.14	2093	1537	1365
245	15	0.30	1670	1678	1639
246	6	0.12	2085	1589	1322
247	10	0.20	2227	1620	1145
248	9	0.18	2166	1520	1307
249	9	0.18	2151	1543	1299
25	22	0.44	1683	1213	2084
250	11	0.22	1925	1494	1572
251	15	0.30	1884	1723	1380
252	7	0.14	2085	1699	1211
253	16	0.32	2072	1553	1361
254	8	0.16	2033	1651	1310
255	14	0.28	2162	1586	1240
256	6	0.12	2113	1365	1518
257	11	0.22	2109	1688	1194
258	7	0.14	2128	1554	1313
259	24	0.48	2042	1482	1454
26	3	0.06	2056	1031	1912
260	9	0.18	2051	1577	1365
261	6	0.12	2154	1525	1317
262	12	0.24	2214	1351	1425
263	6	0.12	2124	1689	1183
264	12	0.24	1846	1604	1540
265	10	0.20	1955	1652	1385
266	13	0.26	2044	1655	1290
267	4844	96.84	3	42	113
268	17	0.34	1597	1942	1446

269	5	0.10	2145	1609	1243
27	4	0.08	1862	906	2230
270	14	0.28	1989	1600	1399
271	15	0.30	1967	1643	1377
272	5	0.10	2101	1616	1280
273	3	0.06	2132	1418	1449
274	7	0.14	2144	1629	1222
275	11	0.22	2065	1551	1375
276	12	0.24	2120	1570	1300
277	7	0.14	1959	1606	1430
278	9	0.18	2001	1543	1449
279	6	0.12	2024	1574	1398
28	7	0.14	1970	1037	1988
280	14	0.28	2031	1582	1375
281	16	0.32	2076	1490	1420
282	10	0.20	2021	1550	1421
283	7	0.14	2006	1661	1328
284	10	0.20	2106	1451	1435
285	15	0.30	2036	1583	1368
286	10	0.20	2043	1435	1514
287	25	0.50	1880	1582	1515
288	6	0.12	1925	1663	1408
289	19	0.38	1804	1741	1438
29	13	0.26	1895	1034	2060
290	19	0.38	2042	1528	1413
291	7	0.14	2049	1524	1422
292	11	0.22	1959	1630	1402
293	7	0.14	1973	1555	1467
294	9	0.18	1458	2049	1486
295	12	0.24	2076	1720	1194
296	8	0.16	2163	1336	1495
297	10	0.20	2025	1497	1470
298	4	0.08	2057	1467	1474
299	10	0.20	2122	1550	1320
30	7	0.14	2056	838	2101
300	5	0.10	2232	1541	1224
31	14	0.28	1815	959	2214
32	8	0.16	1900	845	2249
33	12	0.24	1985	989	2016
34	7	0.14	2223	987	1785
35	3	0.06	1860	990	2149
36	41	0.82	1900	973	2088
37	9	0.18	1949	1081	1963
38	12	0.24	1761	941	2288

39	8	0.16	1898	1045	2051
4	18	0.36	1884	833	2267
40	12	0.24	2052	1017	1921
41	19	0.38	2007	880	2096
42	12	0.24	1962	823	2205
43	20	0.40	2337	957	1688
44	11	0.22	2034	1121	1836
45	2	0.04	2109	834	2057
46	19	0.38	2263	783	1937
47	8	0.16	2041	847	2106
48	9	0.18	1770	958	2265
49	32	0.64	1898	1109	1963
5	19	0.38	1899	928	2156
50	7	0.14	2151	1077	1767
51	14	0.28	1937	1209	1842
52	14	0.28	1846	1118	2024
53	11	0.22	1859	1059	2073
54	7	0.14	1978	1074	1943
55	8	0.16	1991	976	2027
56	10	0.20	1841	850	2301
57	11	0.22	1852	881	2258
58	3	0.06	2113	969	1917
59	11	0.22	1808	1035	2148
6	6	0.12	1971	952	2073
60	16	0.32	2111	853	2022
64	15	0.30	2187	969	1831
65	27	0.54	1589	1184	2202
66	5	0.10	1645	1226	2126
67	18	0.36	1765	1149	2070
68	10	0.20	2358	920	1714
69	7	0.14	1662	1180	2153
7	10	0.20	1843	975	2174
70	7	0.14	2149	1118	1728
71	22	0.44	2272	1071	1637
72	11	0.22	2136	998	1857
73	3	0.06	1901	1018	2080
74	5	0.10	2336	1035	1626
75	3	0.06	2438	883	1678
76	3	0.06	2276	1102	1621
77	17	0.34	2271	889	1825
78	14	0.28	2213	1045	1730
79	11	0.22	2216	1029	1746
8	2	0.04	1996	908	2096
80	8	0.16	2204	890	1900

81	10	0.20	1899	1004	2089
82	31	0.62	1950	900	2121
83	15	0.30	2407	1147	1433
84	6	0.12	2386	1049	1561
85	23	0.46	2478	976	1525
86	6	0.12	1866	962	2168
87	17	0.34	2441	861	1683
88	10	0.20	2001	939	2052
89	6	0.12	1959	954	2083
9	11	0.22	2077	916	1998
90	13	0.26	2339	937	1713
91	8	0.16	2290	1040	1664
92	2	0.04	2362	937	1701
93	4	0.08	2237	998	1763
94	16	0.32	1940	1022	2024
95	12	0.24	2358	877	1755
96	20	0.40	2129	1024	1829
97	12	0.24	2289	999	1702
98	18	0.36	2112	933	1939
99	17	0.34	2287	1028	1670
